# Recurrent neural network for the dynamics of Zika virus spreading

**DOI:** 10.3934/publichealth.2024022

**Published:** 2024-04-02

**Authors:** Kottakkaran Sooppy Nisar, Muhammad Wajahat Anjum, Muhammad Asif Zahoor Raja, Muhammad Shoaib

**Affiliations:** 1 Department of Mathematics, College of Science and Humanities in Al Kharj, Prince Sattam bin Abdulaziz University, 11942, Saudi Arabia; 2 Saveetha School of Engineering, SIMATS, Chennai, India; 3 Department of Mathematics, COMSATS University Islamabad, Attock Campus, Pakistan; 4 Future Technology Research Center, National Yunlin University of Science and Technology, 123 University Road, Section .3, Douliou, Yunlin 64002, Taiwan, R.O.C; 5 Yuan Ze University, AI Center, Taoyuan 320, Taiwan

**Keywords:** recurrent neural networks, SEIR nonlinear system, Zika virus, regression, Adam's method

## Abstract

Recurrent Neural Networks (RNNs), a type of machine learning technique, have recently drawn a lot of interest in numerous fields, including epidemiology. Implementing public health interventions in the field of epidemiology depends on efficient modeling and outbreak prediction. Because RNNs can capture sequential dependencies in data, they have become highly effective tools in this field. In this paper, the use of RNNs in epidemic modeling is examined, with a focus on the extent to which they can handle the inherent temporal dynamics in the spread of diseases. The mathematical representation of epidemics requires taking time-dependent variables into account, such as the rate at which infections spread and the long-term effects of interventions. The goal of this study is to use an intelligent computing solution based on RNNs to provide numerical performances and interpretations for the SEIR nonlinear system based on the propagation of the Zika virus (SEIRS-PZV) model. The four patient dynamics, namely susceptible patients S(y), exposed patients admitted in a hospital E(y), the fraction of infective individuals I(y), and recovered patients R(y), are represented by the epidemic version of the nonlinear system, or the SEIR model. SEIRS-PZV is represented by ordinary differential equations (ODEs), which are then solved by the Adams method using the Mathematica software to generate a dataset. The dataset was used as an output for the RNN to train the model and examine results such as regressions, correlations, error histograms, etc. For RNN, we used 100% to train the model with 15 hidden layers and a delay of 2 seconds. The input for the RNN is a time series sequence from 0 to 5, with a step size of 0.05. In the end, we compared the approximated solution with the exact solution by plotting them on the same graph and generating the absolute error plot for each of the 4 cases of SEIRS-PZV. Predictions made by the model appeared to be become more accurate when the mean squared error (MSE) decreased. An increased fit to the observed data was suggested by this decrease in the MSE, which suggested that the variance between the model's predicted values and the actual values was dropping. A minimal absolute error almost equal to zero was obtained, which further supports the usefulness of the suggested strategy. A small absolute error shows the degree to which the model's predictions matches the ground truth values, thus indicating the level of accuracy and precision for the model's output.

## 1. Introduction

Recurrent neural networks (RNNs) are an advanced class of artificial neural networks that use feedback loops in their design to process sequential data. RNNs are inherently able to extract temporal dependencies and context from previous data, in contrast to traditional feedforward neural networks, which strictly process data sequentially without remembering the previous inputs. Because of this unique quality, RNNs are especially well-suited for sequential data tasks such as speech recognition, handwriting recognition, processing natural languages, and time series prediction. Using their capacity to represent sequential data and capture temporal dependencies, RNNs present a promising method to comprehend and forecast the dynamics of the Zika virus spreading. Traditional epidemiological models may find it difficult to explain the complex interplay of factors that influence the spread of the Zika virus due to the complex and dynamic nature of infectious disease transmission. Since RNNs are excellent at identifying patterns and trends in sequential data, they are a good choice to examine the temporal dynamics of the spread of disease. Using time-series data on variables such as human mobility, environmental conditions, population demographics, and vector abundance, RNNs can be trained to forecast how Zika virus outbreaks will develop over time. The potential of RNNs in modeling and predicting the spread of Zika virus is examined in this introduction, emphasizing the latter's ability to offer insightful information for public health initiatives. The global public health systems have faced substantial challenges as a result of the Zika virus outbreak in recent years. To effectively control and mitigate Zika virus, it is essential to comprehend its dynamics and patterns of transmission. Tracking the virus's spread has been made possible using conventional epidemiological techniques. However, now that sophisticated computational methods such as RNNs are available, we have a chance to improve our comprehension of the temporal dynamics of Zika virus transmission. RNNs are a class of artificial neural networks that are useful for time series analysis tasks because they are especially well-suited for modeling sequential data. RNNs can capture dependencies and temporal patterns found in time series datasets by taking advantage of the sequential nature of the data. RNNs present a promising method to analyze dynamic patterns of infection spread over time in the context of Zika virus transmission. Our goal is to extract patterns and trends in the transmission of Zika virus by applying RNNs to dynamic data. We use RNNs to look for hidden temporal relationships in the data that might not be immediately visible with more conventional analytical techniques. In addition, our goal is to assess how well RNN models predict the dynamics of Zika virus transmission, which will yield important information for public health interventions and decision-making. This study is important because it adds to the expanding corpus of research on machine learning approaches applied to infectious disease epidemiology. We may be able to predict Zika virus transmission patterns, pinpoint high-risk areas, and more efficiently deploy resources to stop the virus's spread by utilizing the power of RNNs. In the end, the results of this study may help develop proactive methods for Zika virus monitoring and management, which would ultimately protect public health globally.

The congenital Zika syndrome (CZS)-causing Zika virus, a mosquito-borne flavivirus, was initially discovered in rhesus monkeys in the Zika woodland of Uganda in 1947 [1]. Between 1964 and 2007 [2]–[5], a few rare human cases were recorded in Africa and Asia without having a significant impact on the subject's health. The first ZIKV-related illness epidemic was discovered in Micronesia in 2007 [6], and an outbreak was discovered in French Polynesia in 2013 that later spread to many Pacific islands. The virus experienced an epidemic breakout in Brazil in 2015 [7]. Late in 2016, the number of cases grew much further and spread to several nations worldwide. However, since 2017 [8], there have been fewer cases of Zika virus infection, and no new epidemic outbreaks have been reported.

Most of the nations in south and central America were affected by this pandemic. In roughly 87 nations and territories, ZIKV is still present in the tropical and subtropical areas, where there has recently been evidence of an endemic mosquito-borne transmission of the virus [9]–[11]. Due to the growth of urban populations, international travel and trade, climate change, and a lack of mosquito control efforts, the number of persons infected with ZIKV has gradually increased over the past few decades. Due to lockdowns and coronavirus illness 2019 (COVID-19) patients clogging the healthcare system, the COVID-19 pandemic is also likely to have led to underreporting.

ZIKV has an 11-kilobase, positive-sense, single-stranded, non-segmented RNA genome. A single open reading frame (ORF) in the genome, flanked by 5′ and 3′ untranslated regions, encodes a single polyprotein that is split into three structural proteins (the capsid (C), pre-membrane/membrane (PrM), and envelope (E)), as well as seven non-structural proteins (NS1, NS2A, NS2B, NS3, NS4A, NS4B, and NS5) [12]. The host furin protease cleaves the prM inside the secretory route during maturation to generate the mature membrane protein (M); then, the protein product (Pr) is released from the virion when it leaves the cell and reaches the extracellular environment, which has a neutral pH value [13],[14]. The primary antigenic component of the virus, E, facilitates receptor binding and the fusion of the viral and cell membranes after its entrance [15],[16]. Most vaccinations target the antigen E because it is the main target for neutralizing antibodies (nAbs) [17]. PrM-E expression in mammalian cells results in non-viral particles (VLPs) that resemble infectious virions in terms of their antigenic properties [18],[19]. Key characteristics of viruses [20], such as their fusogenic activity and the ability to elicit a nAb response, are retained by VLPs.

A revolutionary technology that imitates human intellect is artificial intelligence (AI). Neural networks, which are modeled after the structure of the brain, power AI learning. These networks enable activities such as image recognition [21], language processing [22], and more [23]–[26] by processing input through a network of artificial neurons. Neural networks continue to be a key component of AI evolution, thus driving innovation across sectors. Sabir Z [27] used swarming neural networks in computers to tackle the Zika virus scenario, use. Rubio-Solis A [28] presented the concept of using online extreme learning machines and neural networks to predict the Aedes mosquito larval incidence in Recife (Brazil). Mahalakshmi B [29] predicted zika virus using a cloud-based multilayer perceptron neural network (MLPNN).

RNNs are a kind of neural network created for processing sequential inputs. It excels in tasks such as language modeling and speech recognition thanks to looping connections, which preserve the recollection of previous inputs. Improved pattern recognition in sequences is made possible by variants such as LSTM and GRU that solve long-term dependence concerns. GRU is for gated recurrent unit, while LSTM refers for long short-term memory. Recurrent neural networks (RNNs) of the two types mentioned above are made to process sequential data. Using attention-based recurrent neural networks, one method to predict mutations within the influenza A virus over time was studied by Yin R [30]. Gupta AK [31] predicted the omicron virus on CT scan pictures by employing an integrated extended convolutional and recurrent neural networks approach. RNNs [32] can also be used to predict medication resistance in the human immunodeficiency virus (HIV). Sindhu TN [33] presented a novel method to improve the structure of artificial neural networks for engineering and disease data, which was a decreasing failure rate model. Anum Shafiq Anum [34] investigated breast cancer modeling and survival analyses with artificial neural networks, maximum likelihood estimation, and statistics and performed a comparison with artificial neural network techniques to investigate Darcy-Forchheimer's Tangent hyperbolic flow towards a cylindrical surface, with a focus on Parabolic Trough Solar Collectors [35]. Anum Shafiq concentrated on creating a neural network-based intelligent computing system to simulate the bioconvection flow of a second-grade nanofluid with gyrotactics. Shafiq A [36] designed the numerical treatment of the Darcy-Forchheimer flow of Ree-Eyring fluids with a chemical reaction, examined [37] the significance of EMHD (Electromagnetohydrodynamic) graphene oxide (GO) water ethylene glycol nanofluid flow, examined the activation energy [38] and binary chemical reaction effects in the axisymmetric flow of third-grade nanofluid, and carried out a comparative analysis of the maximum likelihood estimation and artificial neural network modeling to evaluate the electrical component reliability [39]. Neural networks have been used in many different fields by Çolak AB and Shafiq A [40]–[45].

Below are some of the highlights from the computer simulations:

- For the study of a SEIR nonlinear system based on the spread of the Zika virus (SEIRS-PZV), an RNN scheme was utilized.
- The dataset for the RNN was computed using the Mathematica function NDSolve and was output with an input range of 0 to 5.
- With 15 hidden neurons and a 2-second delay, the RNN framework was trained using the whole dataset.
- The overlapping of the acquired obtained and Adams numerical source solutions allowed for the evaluation of the suggested stochastic scheme's correctness.
- The performance analysis and comparison studies of the RNN were confirmed by the error histogram, correlation plot, regression plot, and error autoregression.

The sections for research article are arranged as follows:

- Section 2 discusses the mathematical formulation of the SEIRS-PZV model.
- Section 3 presents the methods and tools used to solve the ODEs representing SEIRS-PZV model.
- Section 4 represents the finding of the RNN and data visualization graphs such as regressions, correlations, etc. A comparison between the numerical approach and the RNN model is also discussed.
- Section 5 of the article concludes the entire findings.

The integration of deep learning is a promising development in the ongoing search for ways to combat the Zika virus. By utilizing state-of-the-art deep learning algorithms on the dynamic Zika virus data, we are able to overcome the constraints of conventional approaches and open up new possibilities in terms of a predictive accuracy and statistical visualization. We gain a deeper understanding of the complex patterns and subtleties of viral dynamics with every algorithmic iteration, thus enabling us to make decisions with previously unheard-of accuracy. As we lead this innovative research, let us seize the opportunities and obstacles that come ahead with unwavering resolve. By working together, we can transform how we perceive and treat infectious diseases and open the door to a time when the Zika virus threat will not stand in the way of world prosperity and health.

We explain the theoretical foundations of our research by delving into the mathematical formulation in Section 2 of this article. We describe our methodology in Section 3, providing an overview of the methodical approach used to address the intricacies present in our problem domain. We endeavor to uncover significant insights and solutions by navigating the complex terrain of our research with great care and experimentation. In the presentation and discussion of our findings in Section 4, we carefully examine the data and assess the results of our approaches. This section facilitates a critical reflection and interpretation, thus allowing the readers to comprehend our findings in the context of our research goals. In Section 5, we finally synthesize the main conclusions, implications, and directions for future research by taking on our collective perspectives.

## 2. Mathematical formulation

The study of mathematics is essential to comprehend and forecast the dynamics of the Zika virus's spread. A methodical framework to examine the intricate interactions between different elements that affect the virus's ability to spread within populations is offered by mathematical models. To simulate the dynamics of transmission over time, these models incorporate parameters such as vector abundance, human mobility patterns, environmental factors, and population demographics. Epidemiologists can evaluate how well intervention strategies, vector control measures, vaccination campaigns, and public health policies to avoid the spread of the Zika virus by using mathematical formulas. Furthermore, mathematical modeling can help scientists identify high-risk regions, forecast probable future outbreaks, and effectively deploy resources to either stop or slow the virus's spread. In general, mathematics is an effective weapon in the fight against the Zika virus because it sheds light on the dynamics of its transmission and directs evidence-based decision-making to reduce the virus's negative effects on public health.

Both vector-to-human and human-to-human transmission are used in this section to carry out both types of infection. The entire population of humans *N_H_*(*y*), susceptible humans *S_H_*(*y*), exposed humans *E_H_*(*y*), infected humans *I_H_*(*y*), and recovered humans *R_H_*(*y*) are the categories used to classify this model. In other words, *N_H_*(*y*) = *S_H_*(*y*) + *E_H_*(*y*) + *I_H_*(*y*) + *R_H_*(*y*). The vector classifications of susceptible *S_V_*(*y*), exposed *E_V_*(*y*), and infected *I_V_*(*y*) (i.e., *N_V_*(*y*) = *S_V_*(*y*) + *E_V_*(*y*) + *I_V_*(*y*)) are used to represent the overall mosquito population. The model's mathematical form is given in Eq. (1)–(7) as follows [46]:

$$S_{H}\prime (y)+\beta_{H}(\rho I_{H}(y)+I_{V}(y))S_{H}(y)+{µ}_{H}S_{H}(y)=A_{H},$$ (1)

$$E_{H}\prime (y)+E_{H}(y)(\chi_{H}+{µ}_{H})=S_{H}(y)\beta_{H}(I_{V}(y)+\rho I_{H}(y)),$$ (2)

$$I_{H}\prime (y)+(\eta +\gamma +{µ}_{H})I_{H}(y)=\chi_{H}E_{H}(y),$$ (3)

$$R_{H}\prime (y)+{µ}_{H}R_{H}(y)=\gamma I_{H}(y),$$ (4)

$$S_{V}\prime (y)+\beta_{V}I_{H}(y)S_{V}(y)+{µ}_{H}S_{V}(y)=A_{V},$$ (5)

$$E_{V}\prime (y)+({µ}_{V}+\delta_{H})E_{V}(y)=\beta_{V}S_{V}(y)I_{H}(y),$$ (6)

$$I_{V}\prime (y)+{µ}_{V}I_{V}(y)=\delta_{V}E_{V}(y),$$ (7)

Equation 1 to 7: ODEs representing SEIRS-PZV.

With initial conditions:

$$S_{H}(0)=k_{1},E_{H}(0)=k_{2},I_{H}(0)=k_{3},R_{H}(0)=k_{4},S_{V}(0)=k_{5},E_{V}(0)=k_{6},I_{V}(0)=k_{7}.$$

By creating mathematical equations that explain the transmission process, mathematics plays a crucial role in understanding the dynamics of the Zika virus spreading. These formulas usually contain variables that indicate which members of a population are susceptible, infected, and recovered (or eliminated), as well as parameters that control the rates of transmission, recovery, and other pertinent variables. Researchers can simulate various Zika virus transmission scenarios by using mathematical modeling, which considers variables such as human movement patterns, vector abundance, and environmental conditions. Through numerical solutions of these equations, epidemiologists are able to forecast the Zika virus's temporal spread, evaluate the effects of different intervention approaches, and pinpoint the critical variables that influence the dynamics of transmission. Additionally, the basic reproduction number (R0), which measures the average number of secondary infections produced by a single infected person in a fully susceptible population, can be estimated using mathematical models. These mathematical understandings are important to direct efforts to stop the spread of the Zika virus and to shape public health policies.

## 3. Solution methodology

Utilizing RNNs in epidemic modeling requires a methodical approach with the goal of accurately predicting the future and capturing the temporal dynamics. First, the preprocessing stage of the data involves careful steps such as gathering data from various sources (e.g., case registries and surveillance reports), and then cleaning the data to remove outliers and missing values. Furthermore, finding pertinent predictors such as environmental and demographic variables heavily depend on feature selection. By organizing the data into suitable time intervals, temporal aggregation improves the data even more and makes model training easier. After dividing the data into training, validation, and test sets, the model parameters are optimized using gradient-based optimization methods such as Adam or RMSprop during the training phase. Regularization strategies such as early stopping and dropout aid in ensuring the generalizability and preventing overfitting. Hyperparameter tuning maximizes the model's predictive power by fine-tuning it even more based on validation performance. There are several crucial steps in the methodology for using RNNs to simulate the Zika virus outbreak that are specific to the epidemic's features. To obtain pertinent epidemiological data about Zika virus cases, demographic factors, environmental conditions, and other relevant variables, it is first necessary to conduct extensive data gathering from a variety of sources, including research studies, public health agencies, and online databases. Following extensive preprocessing, the data is cleaned to remove missing or incorrect values and features selected to find important predictors affecting the dynamics of Zika virus transmission.

**Figure 1.**
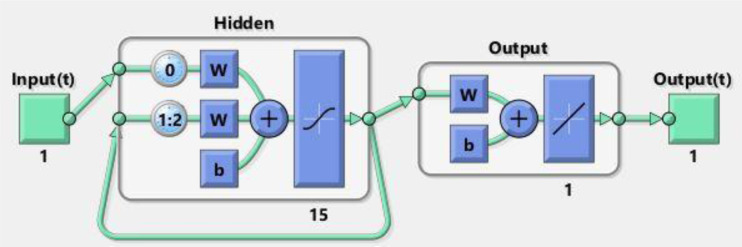
Working Diagram of RNN.

RNNs are made for processing data sequences such as time series and spoken language. It makes use of internal loops to keep track of prior inputs, thus enabling it to recognize relationships and sequential patterns. The LSTM and GRU versions solve this by providing enhanced learning and retention of information over lengthy sequences. However, traditional RNNs can have difficulty with long-term associations. To use an RNN, we first require a dataset of inputs and outputs to train the RNN model on specific hidden layers and delays. We utilize a time series sequence from 0 to 5 divided into 100 intervals as the input for the aforementioned equations, together with 15 hidden layers and a 2 second delay. For the output, we employed the ‘NDsolve’ function in Mathematica to numerically solve ODEs produced by Adam's numerical approach. A total of 4 datasets representing 4 variations of initial conditions of SEIRS-PZV model are individually used to train the RNN model. Table 1 represents the values for each variation in SEIRS-PZV and the working diagram of RNN is described below in Figure 1; moreover, values of each variation are provided in Table 1.

**Table 1.** Variation of SEIRS-PZV.

**Initial conditions**							
**Variation**	*k*_1_	*k*_2_	*k*_3_	*k*_4_	*k*_5_	*k*_6_	*k*_7_
**1**	0.1	0.12	0.14	0.16	0.18	0.2	0.22
**2**	0.12	0.14	0.16	0.18	0.2	0.22	0.24
**3**	0.14	0.16	0.18	0.2	0.22	0.24	0.26
**4**	0.16	0.18	0.2	0.22	0.24	0.26	0.28

**Table 2.** Represent the values for parameter which are constant throughout each variation.

**Parameter**	**Value**	**Parameter**	**Value**
*ρ*	0.14	*β_H_*	0.12
*A_H_*	0.1	*µ_H_*	0.1
*η*	0.15	*β_V_*	0.2
*µ_V_*	0.25	*χ_H_*	0.13
*γ*	0.17	*δ_H_*	0.22
*δ_V_*	0.3		

## 4. Results

A total of 4 variations of initial conditions are observed in this study. The numerical solution of ODEs is generated using the Adams numerical method; then, the dataset is thn fed to the RNN as an output with an input of 0 to 5 divided in 100 intervals and 15 hidden layers with delay of 2 seconds. To train the model, 100% of the dataset is utilized. The results from the RNN are analyzed, such as correlations, regressions, autoregressions, etc.

**Figure 2.**
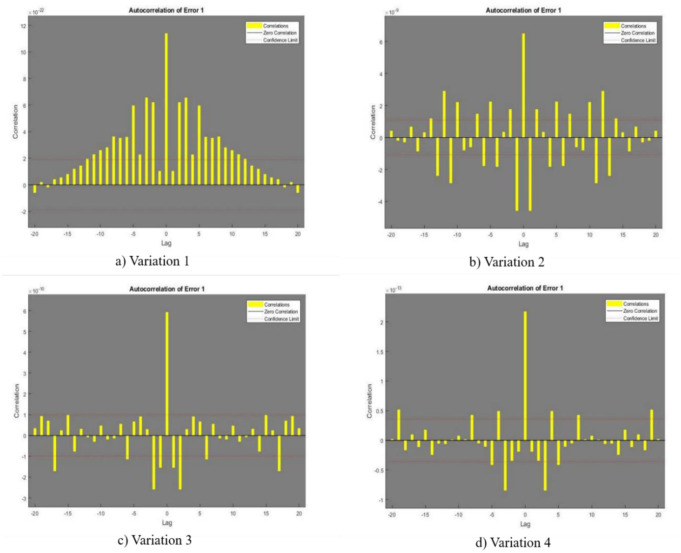
Error Autoregression

**Figure 3.**
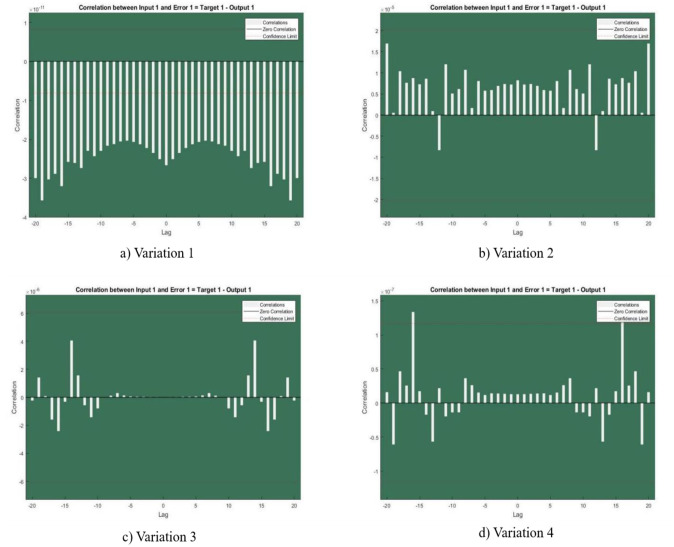
Input Cross-Correlation with Error.

**Figure 4.**
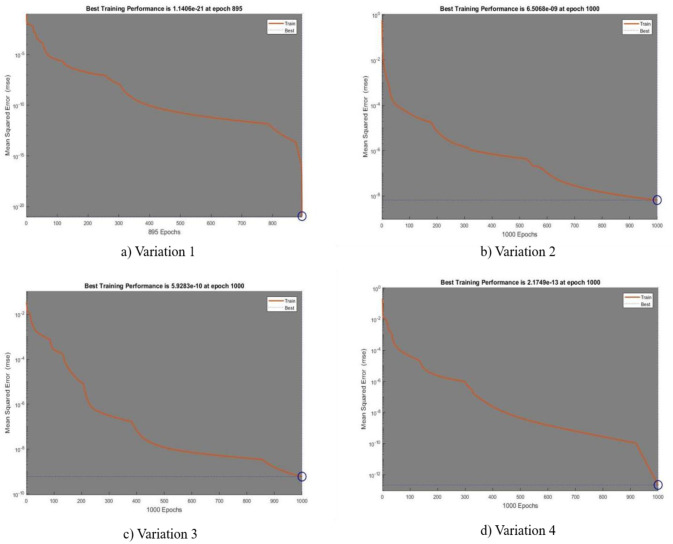
Performance or MSE plot.

**Figure 5.**
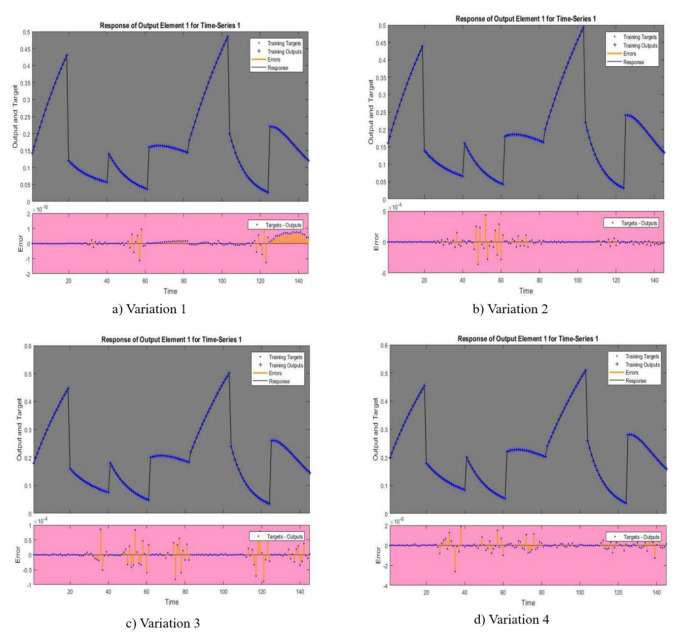
Time series response.

**Figure 6.**
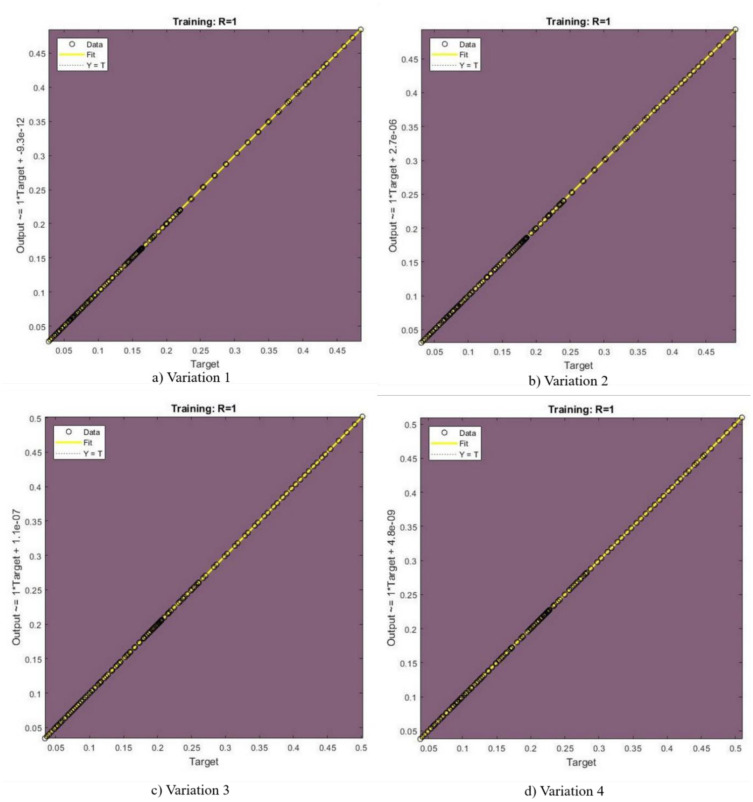
Regression plots.

In Figures 2,3,4,5, and 6, the authors discuss the error autoregression, the input cross-correlation with error, the performance or MSE plot, the time series response, and the regression plots for RNN results, respectively. An important part of time series analysis is the visualization of the error autocorrelation, which sheds light on how residual errors behave over a series of observations. The autocorrelation function (ACF) or partial autocorrelation function (PACF) of the residuals derived from a time series model are usually plotted on error autocorrelation graphs. As diagnostic tools, these graphs help analysts determine whether their models are adequate and help them spot any residual patterns or dependencies in the residuals. An autocorrelation in the residuals is indicated by significant spikes or patterns in the ACF plot at different lags, implying that the model might not be able to fully capture all pertinent information from the data. However, the PACF plot provides additional insights into the underlying structure of the time series by assisting in the isolation of direct relationships between the residuals at various lags. Analysts can improve their models by looking at these graphical representations; they might either add more terms or change some of the parameters to better account for the residual autocorrelation. The intricacies of error autocorrelation graphs are explored in this article, along with their practical applications for time series modeling and forecasting. Analyzing the input cross-correlation with error in time series analysis can reveal important information about the connection between predictor variables and model errors. This graphical representation entails plotting the cross-correlation function (CCF) between the predictors and the residuals from a time series model. Any notable correlations between the predictor variables and the errors can be found by examining the CCF plot; these correlations may point to a bias in the model due to misspecifications or omitted variables. A high correlation between a predictor and the residuals indicates that the predictor's influence may not be fully captured by the model, which could result in biased parameter estimates and forecasts that are erroneous. On the other hand, if predictors and residuals do not correlate, the model might be appropriately specified. It is crucial to comprehend and interpret the input cross-correlation with error graph to improve the time series models, guarantee their accuracy, and make wise forecasting and analysis decisions. The relevance of the input cross-correlation with error graphs, their interpretation, and their usefulness in the time series modeling and forecasting are all examined in this article. A crucial tool to assess the effectiveness of the predictive models is the MSE graph, which is especially useful in a time series analysis and forecasting. This figure illustrates the degree to which a model's predictions match the actual values that were observed at various points in time. Analysts can evaluate the precision and consistency of the model's predictions by charting the MSE over time or across different model specifications. The predictive performance of the model improves when the MSE trend decreases, indicating that the model successfully identifies the underlying patterns in the data. On the other hand, a rising or falling MSE could indicate that the model is having trouble correctly capturing the data, possibly as a result of incorrect specification or insufficient parameterization. A lower MSE suggests that the model's predictions are more accurate. Ultimately, by utilizing the MSE graph, analysts can iteratively improve their predictive models, thus resulting in more accurate forecasts and well-informed decision-making. The relevance of the MSE graph, its interpretation, and its usefulness in evaluating and improving predictive models across a range of fields are all covered in this article. A correlation coefficient (r) of 1 in a linear regression analysis denotes a perfect linear relationship between the response variables and the predictor. This suggests that the response variable proportionately changes and in the same direction for each change in the predictor variable. A perfectly straight line is produced on a graph when the correlation coefficient is 1, meaning that the data points fall exactly along this line, without any deviation. This kind of graph highlights the predictive ability of the linear regression model by showing the strong and positive association between the predictor and response variables. Researchers and analysts must comprehend and interpret this graph to gain insight into the underlying relationship between the variables in the study. It shows the degree to which changes in one variable are mirrored by changes in the other. Additionally, a linear regression correlation coefficient of 1 indicates that the model can correctly predict the response variable from the predictor variable, thus highlighting the applicability of this statistical method in a variety of domains including the social sciences, economics, and engineering.

**Table 3.** Represents the statistical results for RNN.

**Variation**	**Performance**	**Gradient**	**MU**	**Time**	**Final iteration**
**1**	1.14E–21	5.61E–09	1.00E–11	35s	895
**2**	6.51E–09	0.210	1.00E–05	38s	1000
**3**	5.93E–10	2.08E–05	1.00E–07	35s	1000
**4**	2.17E–13	1.93E–05	1.00E–06	40s	1000

**Figure 7.**
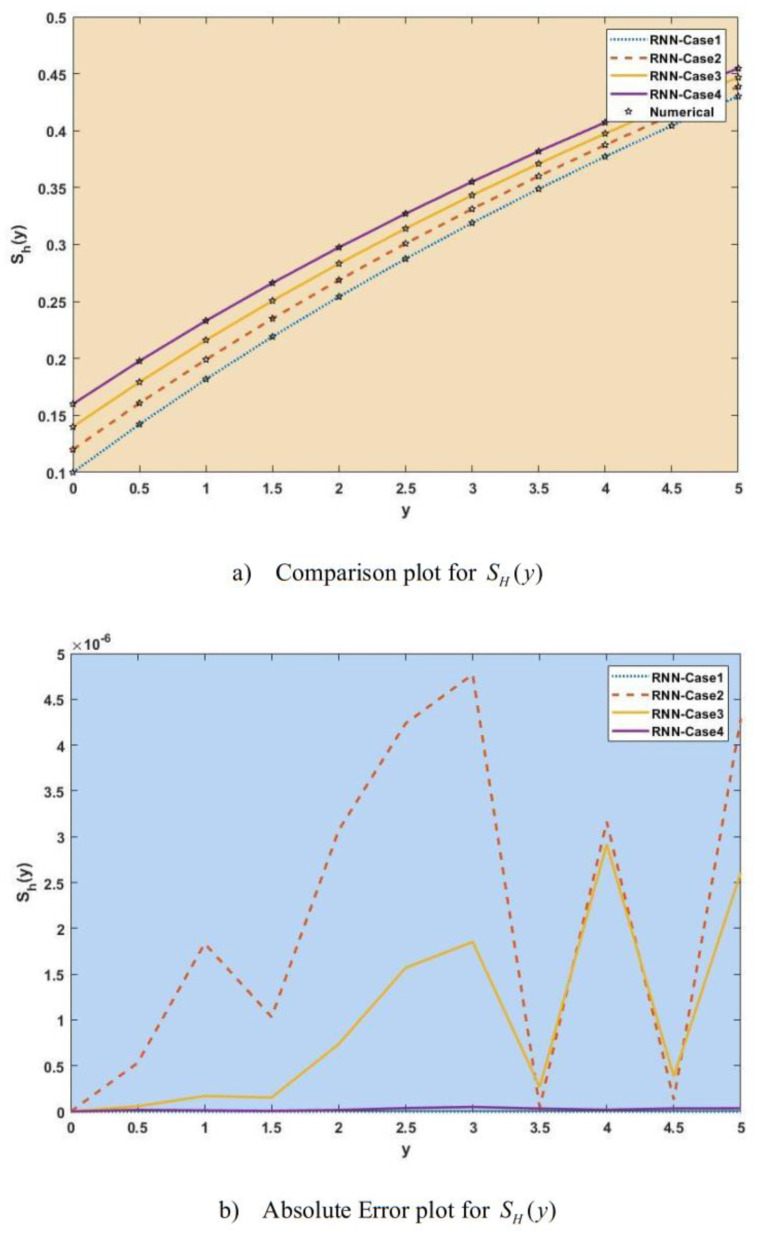
Comparison of RNN with numerical results [*S_H_*(*y*)].

Figure 7–Figure 13 show the result comparison plots and the approximation and numerical (AE) results for the SEIRS-PZV nonlinear system based on the propagation of the Zika virus.

**Figure 8.**
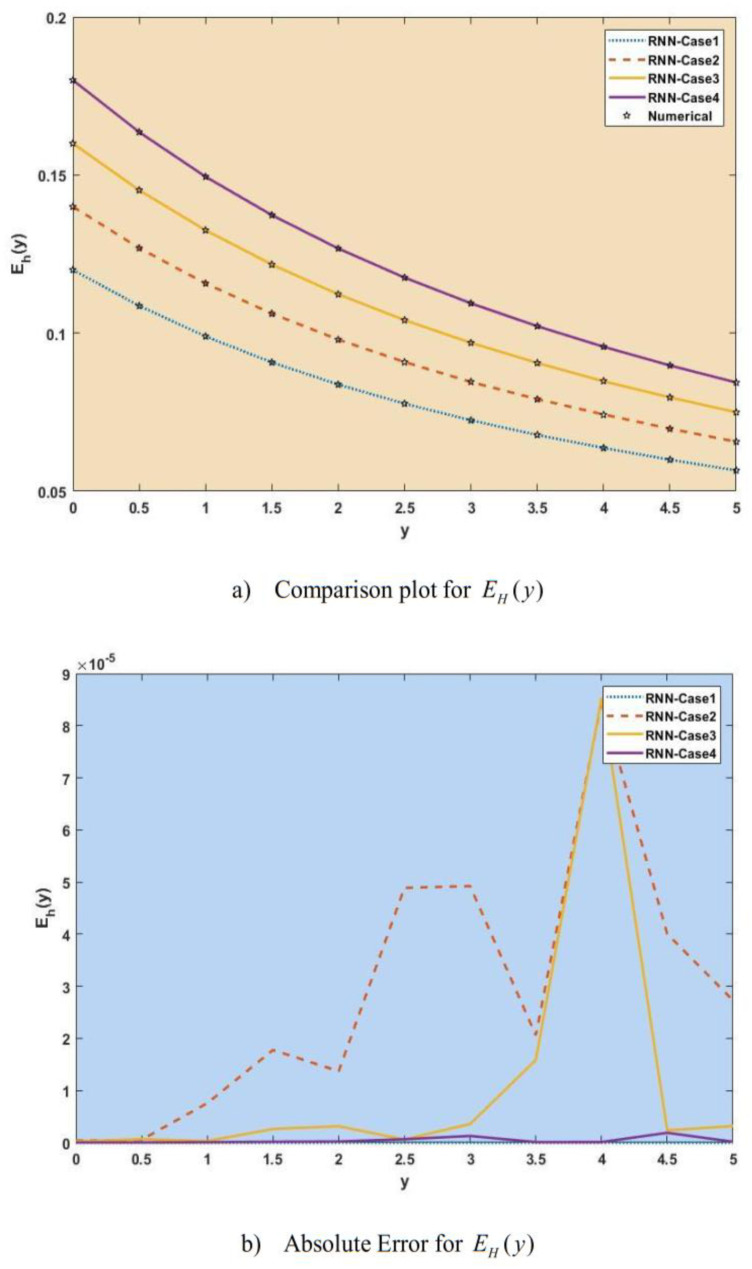
Comparison of RNN with numerical results [*E_H_*(*y*)].

**Figure 9.**
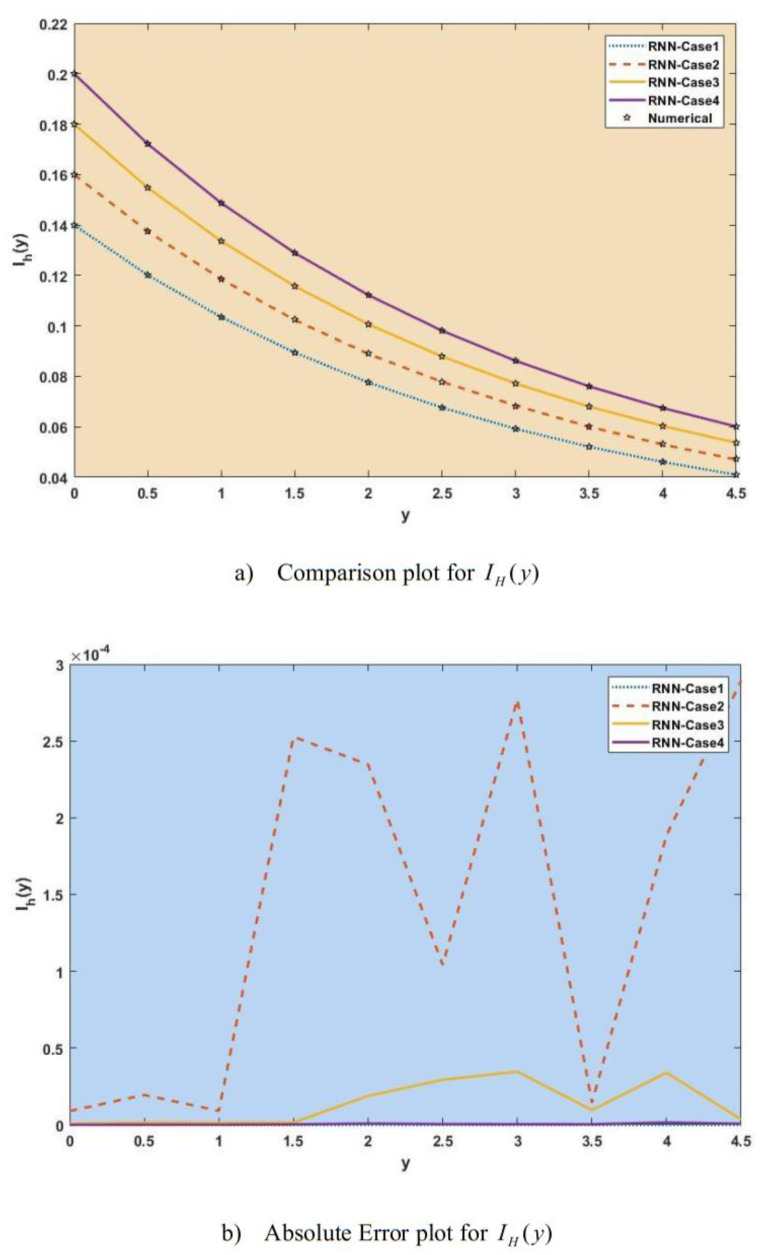
Comparison of RNN with numerical results [*I_H_*(*y*)].

**Figure 10.**
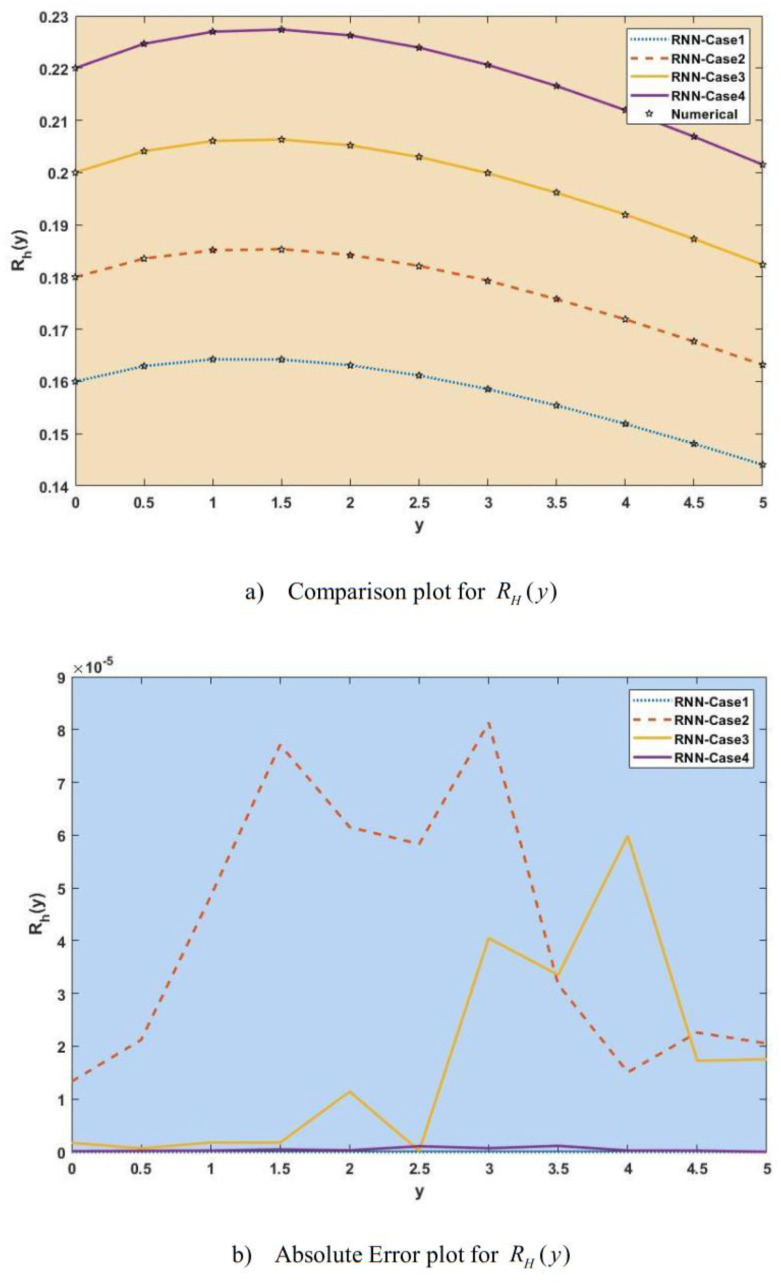
Comparison of RNN with numerical results [*R_H_*(*y*)].

**Figure 11.**
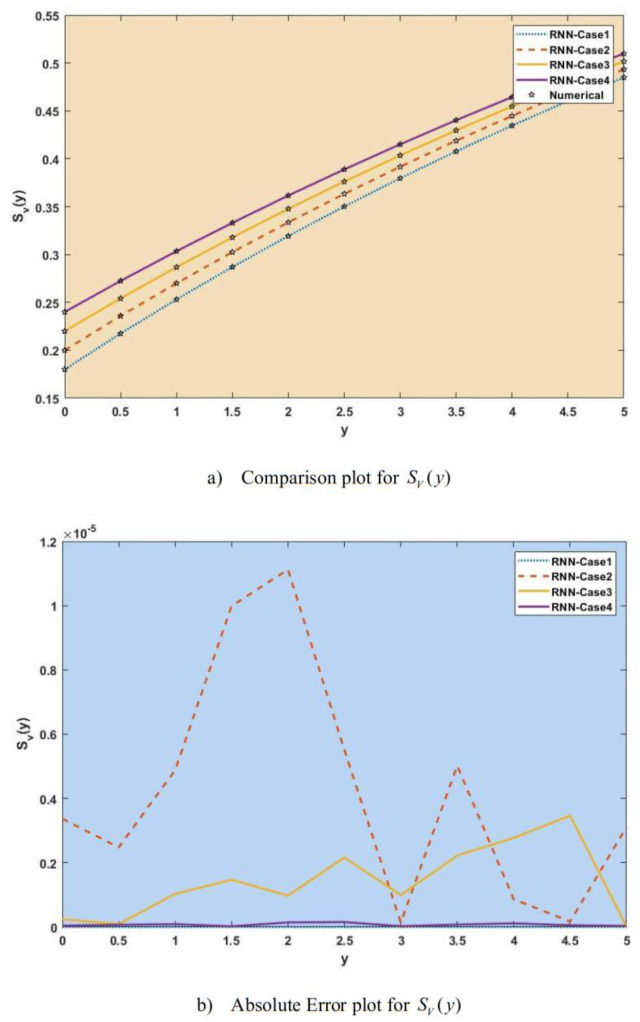
Comparison of RNN with numerical results [*S_V_*(*y*)].

**Figure 12.**
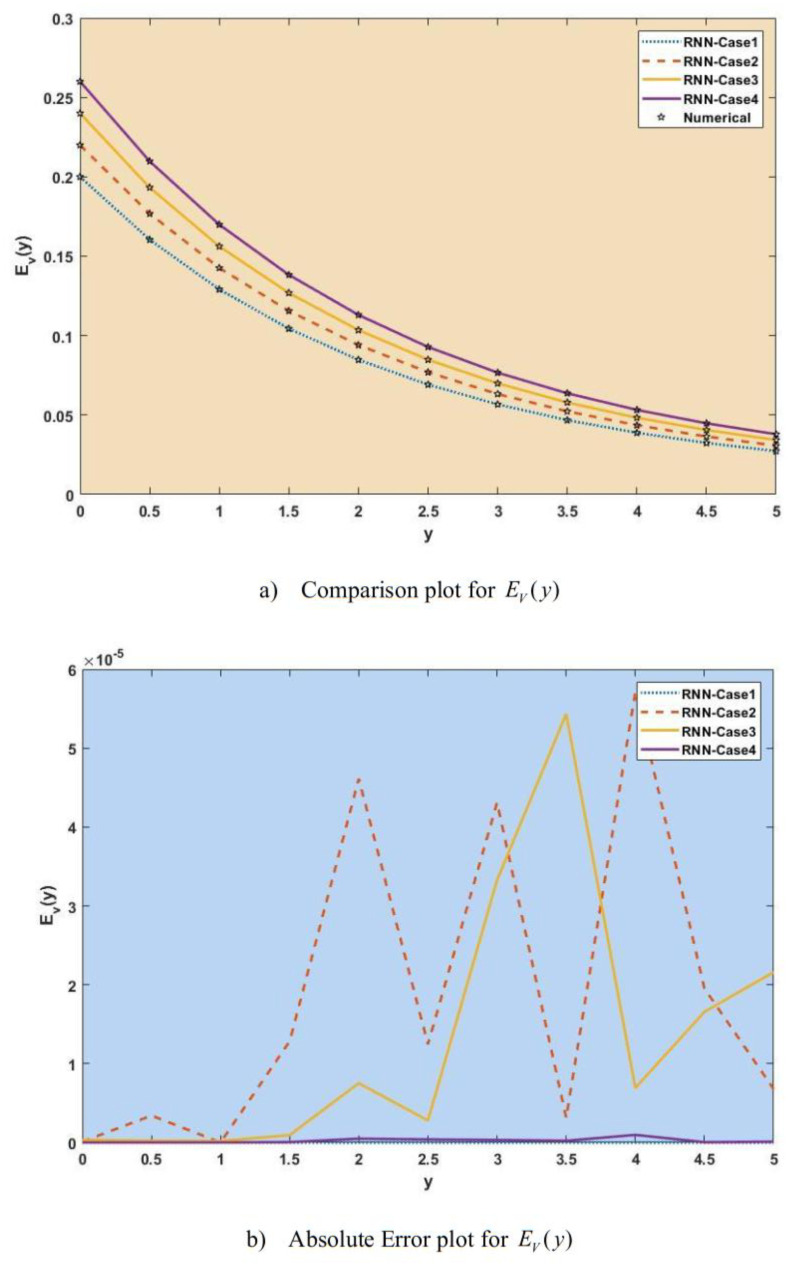
Comparison of RNN with numerical results [*E_V_*(*y*)].

**Figure 13.**
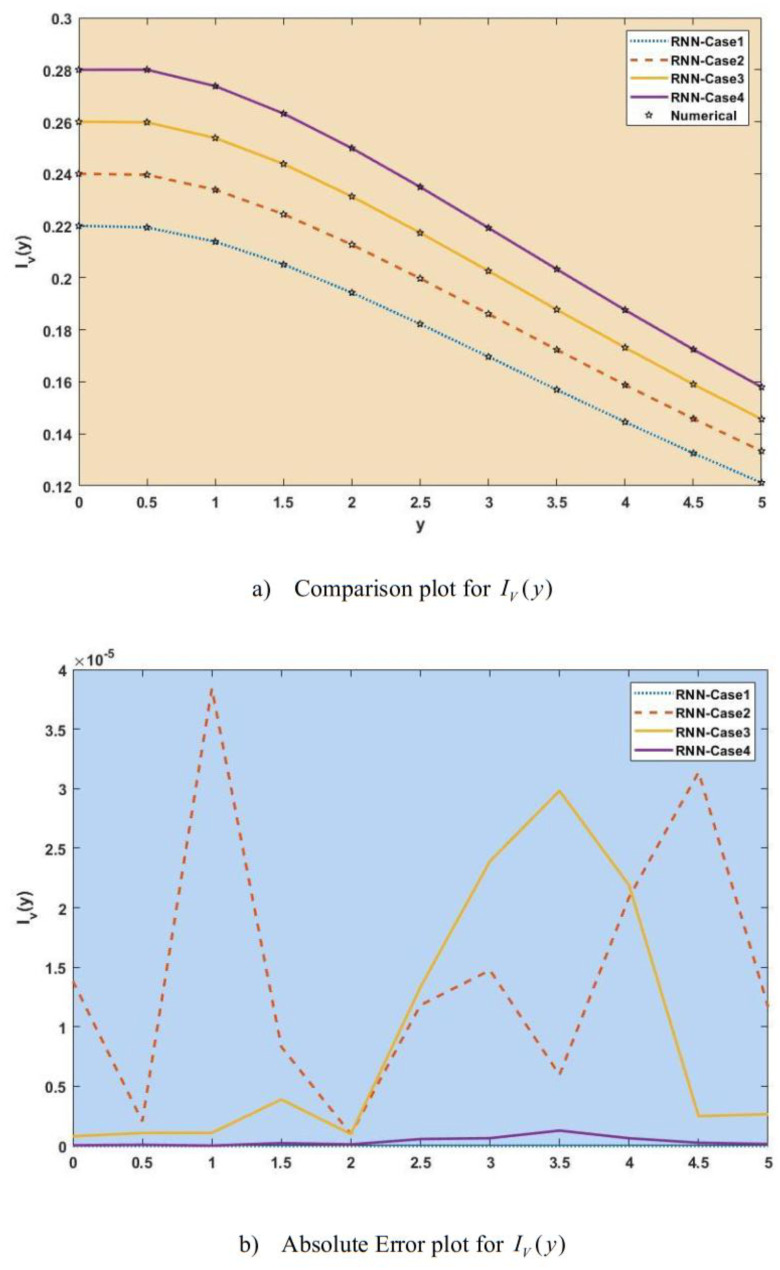
Comparison of RNN with numerical results [*I_V_*(*y*)].

Figures 7a, 8a, 9a, 10a, 11a, 12a, and 13a represent the effect of variation in SEIRS-PZV on *S_H_*(*y*), *E_H_*(*y*), *I_H_*(*y*), *R_H_*(*y*), *S_V_*(*y*), *E_V_*(*y*), and *I_V_*(*y*) profiles, respectively, with an absolute error of SEIRS-PZV in *S_H_*(*y*), *E_H_*(*y*), *I_H_*(*y*), *R_H_*(*y*), *S_V_*(*y*), *E_V_*(*y*), and *I_V_*(*y*) profiles represented by Figures 7b, 8b, 9b, 10b, 11b, 12b, and 13b, respectively. The absolute error for variation 1 and 4 is so low that it's close to 0. Achieving the least amount of absolute error in the data analysis and modeling is a basic goal that shows how accurate and dependable the predictive models are. The relationship between the absolute errors and the values that a model predicts is visually represented by this graphical representation. Reducing the absolute errors—that is, the absolute differences between the observed values and the predicted values—is the aim. If the absolute errors are represented on a graph, the error curve should ideally have a decreasing trend as the predicted values get closer to the true values. This graph helps pinpoint areas where the model might fall short, in addition to showing how predictive it is. To further reduce errors and improve predictive accuracy, analysts can use this data to improve the model, modify parameters, and consider different modeling strategies. In order to assess and enhance the efficacy of predictive models in a variety of industries, such as engineering, healthcare, and finance, it is imperative to comprehend the graphical representation of minimizing absolute error. In order to optimize predictive modeling techniques for improved forecasting accuracy and decision-making, this article explores the subtleties of achieving minimal absolute error, its interpretation, and its practical implications.

## 5. Conclusion

The current study's objective was to provide the numerical results of the SEIRS-PZV nonlinear system based on the RNN approach of Zika virus propagation. RNNs have demonstrated encouraging outcomes when applied to the analysis and prediction of Zika virus data, especially when it comes to obtaining low absolute error, negligible error correlation, and a low MSE. Predictions and an understanding of the disease's spread have become more precise with the use of RNNs in modeling the dynamics of Zika virus transmission. The model's ability to effectively capture the underlying patterns in the data is demonstrated by the low MSE, which showed that the model's predictions closely matched the observed data points. Moreover, the minuscule error correlation indicated that any residual autocorrelation has been effectively captured and addressed by the model, improving its predictive accuracy. Furthermore, the minimal absolute error confirmed the model's accuracy in predicting Zika virus trends, which indicated that the predictions were constantly near to the true values. All things considered, the use of RNNs in Zika virus analysis has a lot of promise to guide public health initiatives and plans to lessen the disease's effects. Further investigation and improvement of RNN-based models can greatly advance our knowledge of the dynamics of the Zika virus and facilitate the creation of preventative measures. The four patient dynamics, namely susceptible patients S(y), exposed patients admitted in a hospital E(y), the fraction of infective individuals I(y), and recovered patients R(y), are represented by the epidemic version of the nonlinear system, or SEIRS-PZV model. The RNN is used to analyze the system's numerical computation capabilities. Here are a few of the study's final observations:

- The RNN approach was used to characterize the numerical depictions of the Zika virus transmission based on the SEIRS-PZV nonlinear system.
- Four different variation that described Zika virus spread were defined using a RNN and numerically stimulated using a specially built computer technique.
- The comparison processes of the acquired reference solutions were used to confirm the accuracy of the scheme.
- 100% of the dataset was used to train the RNN model.
- The data, training objectives, and inputs that have been accessible using the conventional numerical result performances were labeled using the default stoppage limits, step size, and tolerances.
- Using the suggested approach, the AE values of each variation of the SEIRS-PZV nonlinear system of the propagating Zika virus were properly calculated.

## 6. Future direction

Neural Networks (NNs) continue to entice researchers with a multitude of uncharted territories and exciting opportunities in the field of future research. Improving NNs' capabilities and efficiency is becoming more and more important as computational power and technologies grow. To push the limits of what neural networks (NNs) can accomplish, researchers are exploring fields such as deep learning architectures, RNNs, and convolutional neural networks. Furthermore, interdisciplinary applications, where NNs interact with domains such as healthcare, finance, robotics, and environmental science, among others are gaining popularity. NNs are a promising field for future research with a wide range of applications that could spur innovation and advances in many different areas [47]–[52].

## Use of AI tools declaration

The authors declare no Artificial Intelligence (AI) tools have been used in the creation of this article.
